# Framework for automatic information extraction from research papers on nanocrystal devices

**DOI:** 10.3762/bjnano.6.190

**Published:** 2015-09-07

**Authors:** Thaer M Dieb, Masaharu Yoshioka, Shinjiro Hara, Marcus C Newton

**Affiliations:** 1 Graduate School of Information Science and Technology, Hokkaido University, Kita 14, Nishi 9, Kita-ku, Sapporo, Hokkaido, 060-0814, Japan; 2 Research Center for Integrated Quantum Electronics, Hokkaido University, Kita 13, Nishi 8, Sapporo 060-8628, Japan; 3 Physics & Astronomy, University of Southampton, Southampton, SO17 1BJ, UK

**Keywords:** annotated corpus, automatic information extraction, nanocrystal device development, nanoinformatics, text mining

## Abstract

To support nanocrystal device development, we have been working on a computational framework to utilize information in research papers on nanocrystal devices. We developed an annotated corpus called “ NaDev” (*Na*nocrystal *Dev*ice Development) for this purpose. We also proposed an automatic information extraction system called “NaDevEx” (*Na*nocrystal *Dev*ice Automatic Information *Ex*traction Framework). NaDevEx aims at extracting information from research papers on nanocrystal devices using the NaDev corpus and machine-learning techniques. However, the characteristics of NaDevEx were not examined in detail. In this paper, we conduct system evaluation experiments for NaDevEx using the NaDev corpus. We discuss three main issues: system performance, compared with human annotators; the effect of paper type (synthesis or characterization) on system performance; and the effects of domain knowledge features (e.g., a chemical named entity recognition system and list of names of physical quantities) on system performance. We found that overall system performance was 89% in precision and 69% in recall. If we consider identification of terms that intersect with correct terms for the same information category as the correct identification, i.e., loose agreement (in many cases, we can find that appropriate head nouns such as temperature or pressure loosely match between two terms), the overall performance is 95% in precision and 74% in recall. The system performance is almost comparable with results of human annotators for information categories with rich domain knowledge information (source material). However, for other information categories, given the relatively large number of terms that exist only in one paper, recall of individual information categories is not high (39–73%); however, precision is better (75–97%). The average performance for synthesis papers is better than that for characterization papers because of the lack of training examples for characterization papers. Based on these results, we discuss future research plans for improving the performance of the system.

## Introduction

Nanoscale research is a rapidly progressing domain and many research papers containing experimental results have been published. Because it is a very time-consuming task to read through all related papers, several research efforts have been conducted in the nanoinformatics research domain. This includes the construction of databases for sharing the experimental results [1–5], and the set-up of portals for sharing useful information [6–12]. Those approaches try to support data collection processes based on human efforts. It is desirable to have a framework to support information extraction from research papers. This approach is widely used in other research domains. For example, the GENIA corpus [13] was constructed to extract biology-related information (e.g., genome, protein) and the BioCreative IV CHEMDNER corpus [14] was created to extract chemical and drug names. Based on such corpora, several researchers have proposed a variety of methods for the extraction of information from research papers [15–17]. In the nanoinformatics domain, only a few researchers have attempted to automatically extract information from research papers [18–20] and their frameworks are explicitly focused on nanomedicine applications.

Nanocrystal device development [21–26] is an important area of nanoscale research. To support analysis of experimental results in this domain, extracting experimental information from related publications is desirable. We previously constructed an annotated corpus called “NaDev” (*Na*nocrystal *Dev*ice Development corpus) [27–28] for research papers on nanocrystal device development. We also proposed a framework to extract information from research papers by using machine learning tools [29–30]. However, this system was only evaluated using the corpus constructed in our preliminary experiment, which was not sufficient to compare automatic information extraction results with those from human annotators. In addition, in the discussion of constructing NaDev corpus, we found that the paper type (i.e., synthesis or characterization) affected the style of writing, so the information extraction quality varied according to paper type.

In this paper, we propose a framework for automatic information extraction, NaDevEx (*Na*nocrystal *Dev*ice Automatic Information *Ex*traction Framework) from research papers on nanocrystal devices and evaluate the system using the NaDev corpus. Furthermore, we discuss the quality of automatic information extraction compared with that from human annotators and conduct a failure analysis to identify future research issues. In this analysis, we compare the results for synthesis papers with the results for characterization papers to better understand the effect of the type of paper on the system performance.

Before discussing our automatic information extraction experiments using NaDev, we briefly review previous studies on extracting useful information from research papers in other domains and introduce our proposed system for automatic information extraction.

Utilizing information in research papers using text-mining techniques is an increasingly important trend in several domains. In bioinformatics for example, several frameworks for automatic extraction of biomedical entities from research papers have been proposed [15–16]. In the chemical information domain, different approaches compete to extract chemical entities and drug names automatically from the literature [17] using the BioCreative IV CHEMDNER corpus [14]. We can classify approaches to information extraction and named entity recognition into two groups. One is a machine-learning approach that uses a domain corpus, such as GENIA, to find typical patterns for explaining useful terms. The other is a rule-based system that uses rules to extract useful terms (e.g., use a list of chemical symbols to identify chemical compounds). Many recent systems have used a combination of both approaches.

For extracting information from nanocrystal device papers, we have proposed an automatic information extraction framework [29] using machine learning techniques. This approach tries to extract information step-by-step. We call this step-by-step extraction “cascading style extraction” [31].

A preliminary performance check of the automatic information extraction system using the corpus developed for the preliminary experiment confirmed the appropriateness of the general framework. However, the characteristics of NaDevEx were not fully examined. In this paper, we conduct system evaluation experiments for NaDevEx using the NaDev corpus and analyze system performance compared with human annotators’ results. We also discuss plans for future research based on this analysis.

## Materials and Methods

### NaDev corpus

The NaDev corpus [27–28] was constructed to identify experimental information for extraction from nanocrystal device development papers. In order to extract wide varieties of experimental information, NaDev corpus uses full text of research papers instead of abstracts that are commonly used for constructing such corpora. Abstracts usually do not contain detailed explanation about experimental parameters in relation with output evaluation. It is necessary to extract such information to analyze experimental results adequately. In this corpus, eight information categories are annotated as useful information in papers related to nanocrystal device development. These information categories are defined as below:

- Source material (SMaterial): Material used as input in the experiment, such as InGaAs.
- Material characteristic feature (MChar): Characteristic feature of the materials, such as hexagonal. Such feature might be a result of manufacturing process or is a characteristic feature of source material.
- Experimental parameter (ExP): Parameter for controlling experiment’s conditions, such as diameter or total pressure.
- Experimental parameter value (ExPVal): Value of an experimental parameter, such as 50 nm or 10 atoms.
- Evaluation parameter (EvP): Parameter that is used to evaluate the output of the experiment, such as peak energy.
- Evaluation parameter value (EvPVal): Value of an evaluation parameter, such as 1.22 eV.
- Manufacturing method (MMethod): Method used in the experiment to achieve the desired product, such as selective-area metalorganic vapor-phase epitaxy.
- Target artifact or final product (TArtifact): Final output of the experiment, such as nanowires.

**Figure 1:**
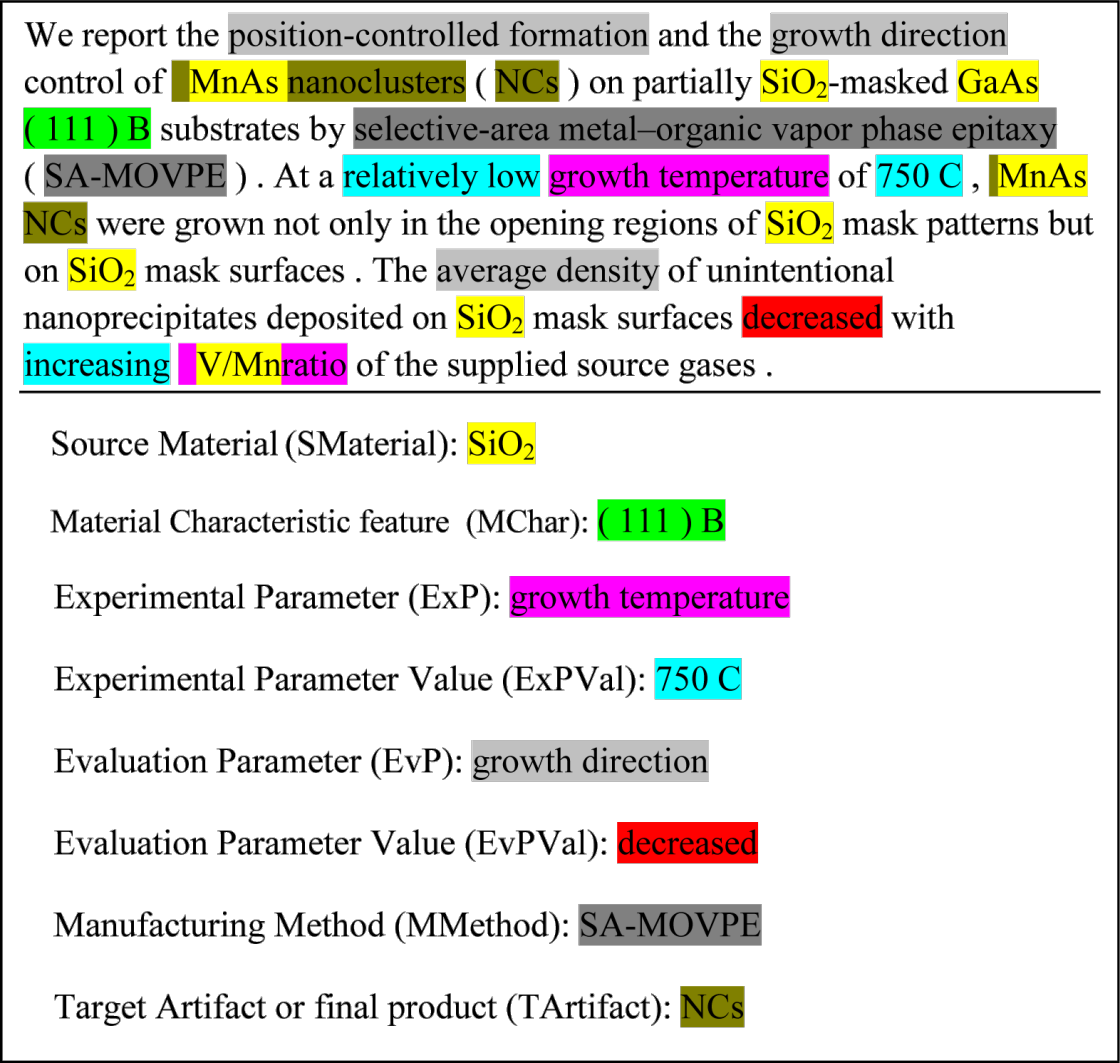
Sample of NaDev corpus.

The NaDev corpus has 392 sentences. 2870 terms are annotated using these information categories. Figure 1 shows a sample of the corpus. Table 1 shows the number of categorized terms in NaDev corpus.

**Table 1:** Number of categorized terms in NaDev corpus.

Information category	SMaterial	MMethod	MChar	TArtifact	ExP	EvP	ExPVal	EvPVal	Total

terms	780	136	381	416	262	365	234	296	2870
of total	27%	5%	13%	15%	9%	13%	8%	10%	

### Corpus construction

**Figure 2:**
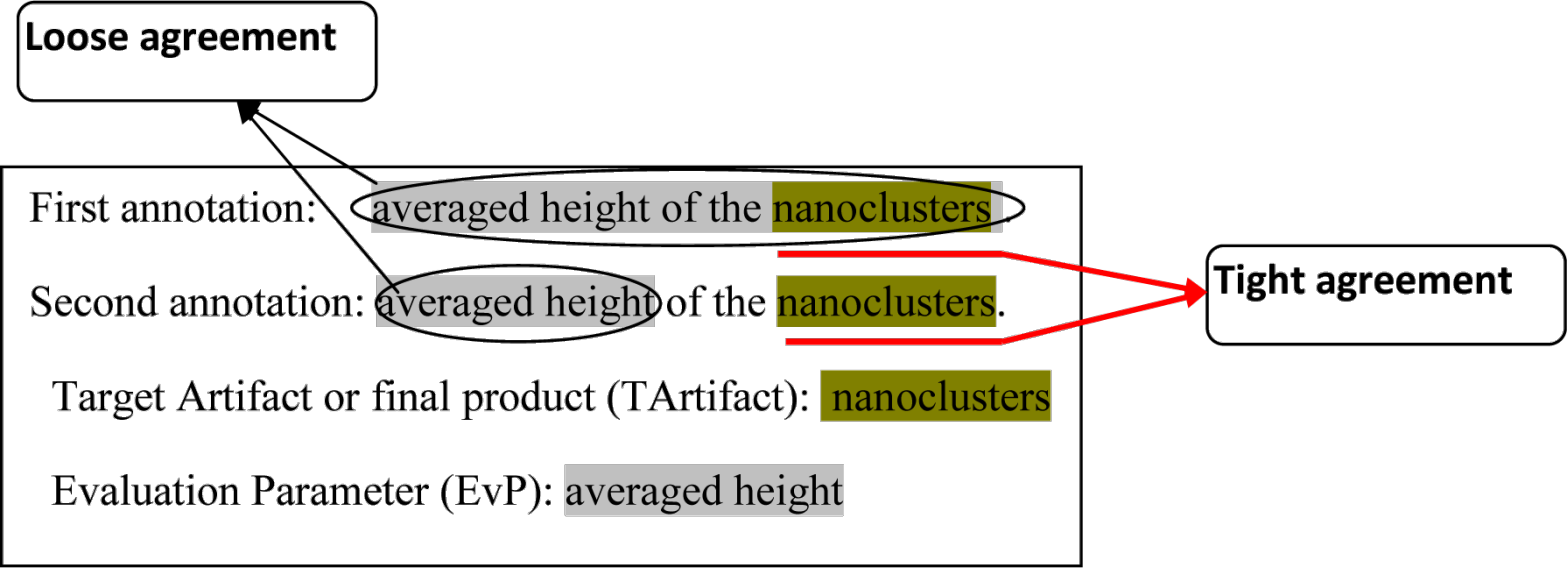
Example of tight and loose agreement.

The corpus construction guideline [27] was prepared in collaboration with a domain expert in nanocrystal device development by using the results of the annotation experiments by domain graduate students. In each experiment, two graduate students were asked to annotate the same paper independently. Annotated results were compared to check the reliability of the guideline. We used kappa coefficient to test inter-annotator agreement (IAA) [32]. Two metrics were used for the analysis: tight agreement, which considers the term boundary and term category to decide the agreement; and loose agreement, which ignores the term boundary, i.e., when a term overlaps with a correct term of the same information category, we treat it as correct (see Figure 2 for an example).

For the inter-annotator mismatch cases, we had meetings for discussing these cases with the annotators, and collected adequate annotation examples for further reference. Inter annotator mismatches, in most cases occurred due to the difficulty to set correct boundaries of the term, specially, in the EvPVal and ExP information categories.

### Corpus evaluation

Even though the corpus construction guideline reached a reliable level with loose agreement [29], it was necessary to evaluate this corpus and finalize it with a domain expert researcher to ensure reliability. We classified the annotations of graduate students into agreed and disagreed annotations. Careless mistakes, such as one annotator missed to add an annotation, or typical types of disagreement when annotators misunderstood the guideline, were easily checked in the discussion after each annotation experiment, so they were considered to be agreed annotations.

To improve the consistency of the annotation and to overcome problems found by examining the corpus, the domain expert proposed few modifications to the corpus-construction guideline.

With the revision of the domain expert, we found the corpus contains two types of papers depending on the content and the writing style. Four of the papers focus on the synthesis of new nanomaterials [33–36], and the other focuses on the characterization of nanomaterials [37]. We have made a finalized version of the five papers of the corpus based on the revision of the domain expert. To evaluate the annotation reliability of the graduate students, we compared this finalized version with the original corpus constructed before the evaluation experiment. Evaluation showed that, if we exclude the effect of the guideline modifications made by the domain expert, for synthesis papers, the agreed annotation results obtained through discussion after the annotation experiments have high precision for all information categories (ranging between 96% and 100%). Discussion between annotators after the annotation process is important, because it can resolve mismatches caused by careless mistakes or misunderstanding of the guideline. Recall is also high (ranging between 91% and 100%). For the characterization paper, the precision is high (ranging between 94% and 100%), but the recall is low because of the larger number of disagreed annotations in this case. The lack of deep domain knowledge of the students for the characterization paper seems to have had a considerable effect on the quality of the annotation.

We concluded generally that information categories such as SMaterial, MMethod, and ExPVal tend to be easier to annotate. Conversely, information categories such as the parameters ExP, and EvP, and EvPVal tend to be more difficult to annotate, requiring deeper domain knowledge, particularly for the characterization paper. Most of the disagreed annotations in these categories resulted from difficulties in setting correct boundaries for these information categories.

### Automatic information extraction

**Figure 3:**
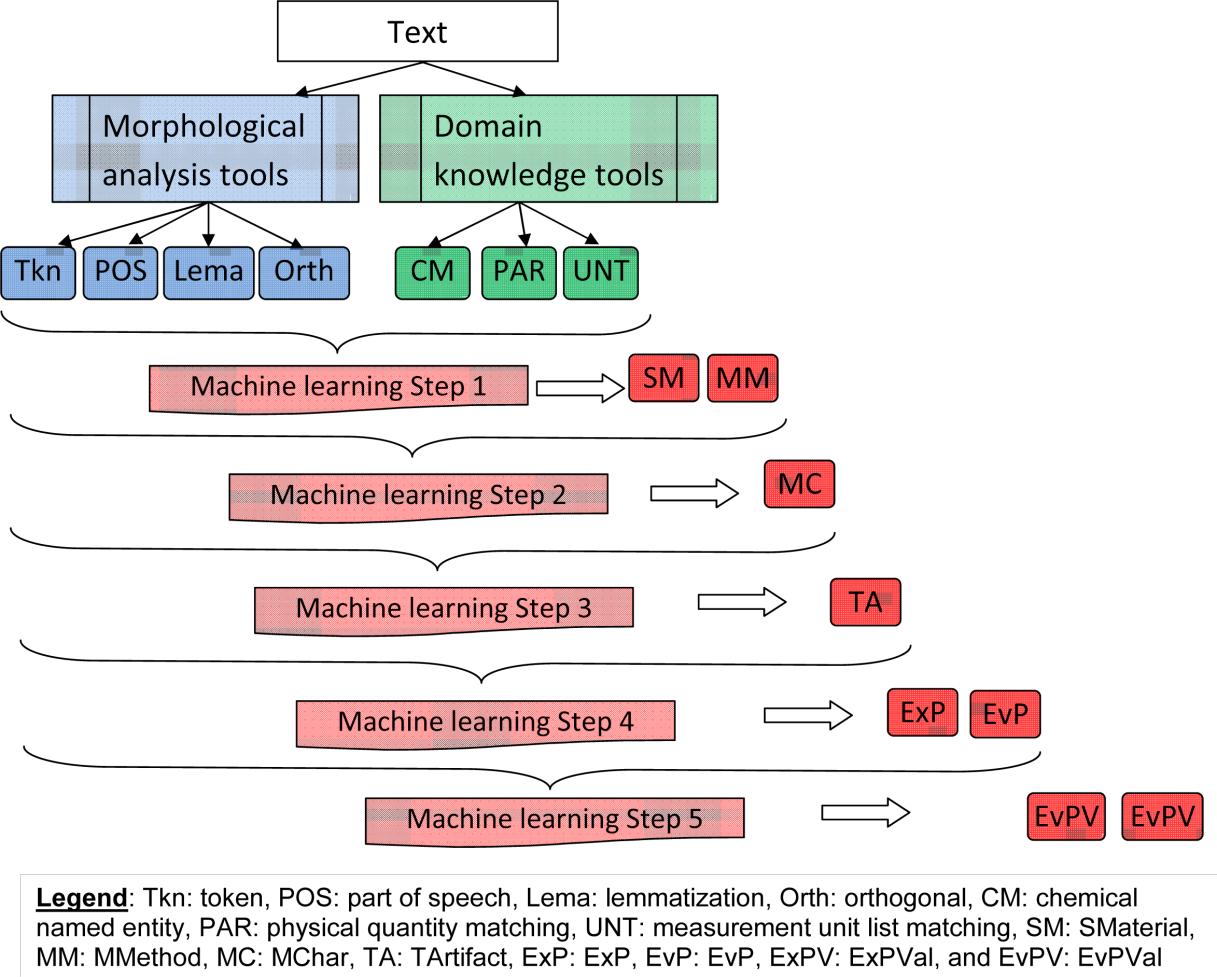
Outline of our automatic information extraction system.

Our information extraction system uses a cascading style extraction based on machine learning. For example, chemical named entities are useful for identifying source materials (e.g., As), and identification of source material is useful for identifying term boundaries of experimental parameters (e.g., pressure of AsH_3_ gas). The order of information categories for extraction was designed by using the overlapping structure between information categories. For example, for experimental parameters and source materials (e.g., pressure of AsH_3_ gas), the extraction of source material should be prior to extraction of experimental parameters. Figure 3 shows a procedure to extract these information categories step-by-step.

First, linguistic features such as part-of-speech (POS) tags, orthogonal features, and lemmatization features are generated using the results from a morphological analysis tool [38]. Second, we use domain knowledge tools (i.e., the output of a chemical named entity recognition tool [29], matching results from a physical quantities vocabulary list, and a list of common measurement units [30]) to generate domain knowledge-related features (CNER, PAR, and UNT, respectively). For the latter step, we used CRF++ [39], an implementation of conditional random field (CRF) [40] as a machine learning system that uses part of the corpus as training data for information extraction. In each step, we use all the features generated by the tools, including linguistic features and domain knowledge-related features.

## Results and Discussion

### System implementation

The NaDevEx system accepts plain text as input and adds annotations to the terms in the text that belong to the information categories defined in the NaDev corpus construction guideline.

Information about the most recent version of the system, which was used for these experiments, is as follows.

- Linguistic features: GPostLL tagger (ver. 0.9.3) [38].
- An orthogonal feature was added using regular expressions based on the definition in [15].
- Domain knowledge-based features: (i) A chemical named entity feature was added using SERB-CNER (Syntactically Enhanced Rule-Based Chemical Named Entity Recognition System) that we developed to annotate chemical entities in nanocrystal device papers. (ii) A parameter identification feature was added based on a list of physical quantities: we compiled a list that contains physical properties of matter (e.g., density, concentration), common parameters found in nanocrystal device papers (e.g., height, conductivity), and several keywords that usually correlate with parameters (e.g., ratio, rate). The list was checked by nanocrystal device researchers as a basic list for physical quantities. (iii) A parameter value identification feature was added based on a list of common measurement units.
- CRF tool: CRF++ (ver.0.58)

**Figure 4:**
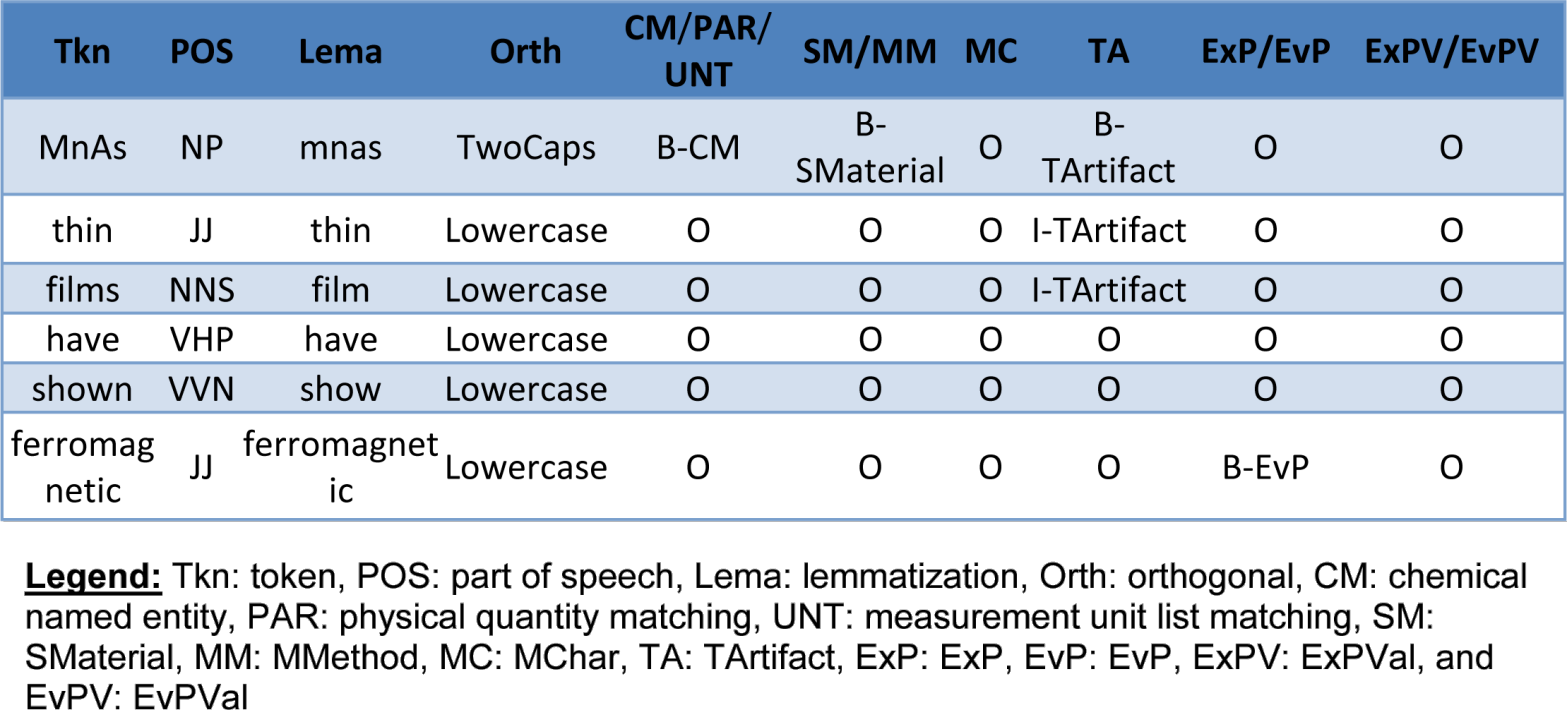
Example of CRF++ input data.

The input for the CRF++ tool is in IOB format, which identifies the position (beginning, inside, out of) of a token of text related to a term. Figure 4 shows an example of input data for the CRF++ tool.

For the training, NaDevEx first added linguistic features and results of the domain knowledge-based systems to the original texts. Then information about correct annotations was used to train the machine learning system CRF++ in cascading style. For the information extraction, the system used the same tools to add linguistic features and results of domain knowledge and used the learning results of CRF++ in cascading style to generate the final answer.

### Experiment plan

In this paper, we evaluate our automatic information extraction system (NaDevEx) and discuss the characteristics of this system by using the NaDev corpus. We design an experiment plan to address the following three main issues:

- system performance analysis compared with human annotators
- system performance analysis for each type of corpus paper (synthesis or characterization)
- effect of domain knowledge features on system performance

### System performance analysis compared with human annotators

We evaluated our system performance using the NaDev corpus. We used five-fold cross validation and calculated precision, recall, and F-score. In each fold, we trained the system using four of the five papers as training data and evaluated its performance using the fifth paper. Because NaDev gold standards are based on the annotation of the domain expert, those results represent the comparison between NaDevEx performance and the annotation of the domain expert. Because NaDevEx is built using machine-learning techniques, deep domain knowledge is difficult to acquire using NaDevEx. Therefore, we contrast NaDevEx performance with that based on agreement between two novice annotators, as discussed previously. These comparison results represent the ideal level of annotation without deep domain knowledge.

Table 2 contrasts the average performance for each information category between NaDevEx and the human annotation results compared with the annotation of the domain expert. Underlining indicates that the difference between NaDevEx performance and the human annotation results is statistically insignificant at the 5% level (*P*≥ 0.05). The human annotations were made prior to the released version of the guideline [27]. Recall of categories that were subject to new definitions (SMaterial and MChar) is underestimated. If we assume that all the new added annotations based on the released guideline were identified by human annotators, recall of SMaterial and MChar is increased to 0.99 and 0.93, respectively.

**Table 2:** Average performance of NaDevEx and the human annotation results compared with the annotation of the domain expert.

	human			NaDevEx		
	precision	recall	F-score	precision	recall	F-score

SMaterial	0.97	0.79	0.87	0.95	0.94	0.94
MMethod	1.00	0.91	0.95	0.97	0.73	0.82
MChar	0.93	0.84	0.88	0.94	0.67	0.75
TArtifact	0.99	0.90	0.94	0.88	0.73	0.80
ExP	1.00	0.91	0.94	0.93	0.68	0.76
EvP	0.98	0.91	0.94	0.78	0.55	0.64
ExPVal	0.99	0.97	0.98	0.80	0.53	0.64
EvPVal	1.00	0.86	0.92	0.75	0.39	0.51
Total	0.98	0.86	0.91	0.89	0.69	0.77

From Table 2, the performance of NaDevEx on the SMaterial category is almost comparable with human annotation. For MMethod, MChar, and ExP, performance is comparatively good for precision but not so good for recall. For the other categories, the system performance is not so good for precision and worse for recall. Based on the nature of the machine-learning system, it is easier to extract the terms that appear in the training data than ones that are unique in the test data. However, if there are similar terms (e.g., a term that overlap with one in the training data or terms used in similar context) in the training data, the system can extract such terms.

There are several cases that show the term boundary identification problem, especially for unique compound terms. To check the effect of such problems, we used the loose agreement metric as illustrated in Figure 2.

For human annotators, even though there were many cases of loose agreement between the two annotators, discussion after annotation experiments generally resolved these boundary mismatch issues. Table 3 contrasts the average performance for each information category for NaDevEx and the human annotation results for loose agreement compared with the annotation of the domain expert. Underlining indicates that the difference between NaDevEx performance and the human annotation results is statistically insignificant at the 5% level (*P*≥ 0.05).

**Table 3:** Average performance of NaDevEx and the human annotation results for loose agreement compared with the annotation of the domain expert.

	human			NaDevEx		
	precision	recall	F-score	precision	recall	F-score

SMaterial	0.99	0.81	0.89	0.98	0.97	0.97
MMethod	1.00	0.91	0.95	0.98	0.73	0.83
MChar	0.94	0.85	0.89	0.96	0.68	0.77
TArtifact	1.00	0.90	0.95	0.96	0.79	0.86
ExP	1.00	0.91	0.95	0.97	0.71	0.79
EvP	0.99	0.92	0.95	0.86	0.60	0.71
ExPVal	1.00	0.97	0.99	0.92	0.62	0.74
EvPVal	1.00	0.86	0.92	0.88	0.46	0.60
Total	0.99	0.87	0.92	0.95	0.74	0.83

The differences between the evaluation results of Table 2 and Table 3 reflect the difficulty of identifying term boundaries. For NaDevEx, performance for loose agreement improves for all information categories in precision and recall, especially for TArtifact, EvP, ExPVal, and EvPVal. This shows that these categories have many problems related to identifying term boundaries. If we accept loose agreement as correct (in most cases we can find appropriate head nouns such as temperature, or pressure in loose matching terms), TArtifact and EvPVal also become almost comparable with human annotation for precision.

In general, Table 2 and Table 3 show that NaDevEx has problems in identifying term boundaries in categories where human annotators have the same difficulty. However, discussion between the annotators after each annotation experiment helped to reduce these difficulties.

In addition, recall of the categories MChar, ExP, EvP, ExPVal, and EvPVal is comparatively worse than that made by the human agreement. For these categories, there are varieties of compound terms that usually contain characteristic technical terms within their boundaries. However, because of the variability in using these technical terms for constructing compound terms, NaDevEx cannot extract such terms appropriately. We discuss this issue in detail in the section “Effect of domain knowledge features on system performance”.

### System performance analysis based on type of paper

System performance differs between synthesis papers and characterization papers. Table 4 shows the average performance of NaDevEx for four synthesis papers and one characterization paper including loose agreement cases using five-fold cross validation.

**Table 4:** NaDevEx average performance on synthesis and characterization papers using five-fold cross validation.^a^

	average synthesis papers						characterization paper					
	prec	rec	F	L-prec	L-rec	F	prec	rec	F	L-prec	L-rec	F

SMaterial	0.95	0.94	0.94	0.98	0.97	0.97	0.93	0.96	0.95	0.96	0.99	0.97
MMethod	0.97	0.75	0.84	0.98	0.76	0.85	1.00	0.63	0.77	1.00	0.63	0.77
MChar	0.94	0.78	0.85	0.96	0.79	0.86	0.92	0.22	0.36	1.00	0.24	0.39
TArtifact	0.93	0.79	0.85	0.95	0.81	0.87	0.69	0.49	0.57	1.00	0.71	0.83
ExP	0.91	0.77	0.83	0.96	0.81	0.87	1.00	0.31	0.48	1.00	0.31	0.48
EvP	0.80	0.57	0.66	0.88	0.62	0.73	0.73	0.48	0.58	0.77	0.51	0.61
ExPVal	0.81	0.57	0.66	0.95	0.67	0.78	0.76	0.41	0.53	0.82	0.44	0.57
EvPVal	0.74	0.41	0.53	0.87	0.48	0.62	0.79	0.33	0.46	0.90	0.37	0.53
Total	0.90	0.75	0.82	0.95	0.79	0.86	0.82	0.47	0.60	0.93	0.53	0.68

^a^prec: precision, rec: recall, L-prec: loose precision, L-rec: loose recall, F: F-score

One reason for the lower performance with the characterization paper is a lack of examples of sentences and terms that are frequently used in characterization papers and not in synthesis papers. To discuss this effect, we conducted a 10-fold cross validation that uses four papers and half of the fifth paper as training data, evaluated on the other half of the fifth paper. Table 5 shows the average performance of NaDevEx on four synthesis papers and one characterization paper using 10-fold cross validation including loose agreement.

**Table 5:** NaDevEx average performance on synthesis and characterization papers using 10-fold cross validation.^a^

	average synthesis papers						average characterization paper					
	prec	rec	F	L-prec	L-rec	F	prec	rec	F	L-prec	L-rec	F

SMaterial	0.95	0.94	0.94	0.98	0.97	0.97	0.96	0.97	0.96	0.97	0.99	0.98
MMethod	0.96	0.81	0.87	0.96	0.81	0.87	1.00	0.63	0.77	1.00	0.63	0.77
MChar	0.95	0.83	0.89	0.97	0.84	0.90	0.84	0.35	0.46	0.87	0.37	0.49
TArtifact	0.95	0.85	0.90	0.96	0.87	0.91	0.71	0.53	0.61	0.98	0.75	0.85
ExP	0.93	0.81	0.86	0.98	0.86	0.91	0.59	0.33	0.42	0.88	0.46	0.61
EvP	0.80	0.63	0.70	0.88	0.69	0.77	0.77	0.47	0.58	0.87	0.53	0.66
ExPVal	0.81	0.67	0.73	0.93	0.77	0.83	0.69	0.46	0.55	0.78	0.51	0.61
EvPVal	0.75	0.48	0.58	0.88	0.56	0.68	0.78	0.35	0.48	0.93	0.41	0.57
Total	0.91	0.79	0.84	0.96	0.83	0.89	0.80	0.51	0.62	0.93	0.59	0.72

^a^prec: precision, rec: recall, L-prec: loose precision, L-rec: loose recall, F: F-score

In this case, because we can use one-half of a paper as training data, the number of terms that are unique to the test data decreased. The performance for 10-fold cross validation is slightly better than that for five-fold cross validation. However, in total, the increased ratio for characterization with loose recall was slightly better than that for synthesis papers.

### Effect of domain knowledge features on system performance

As we have already discussed, it is difficult for the machine learning system to find terms that are unique to the test data. Table 6 shows the number of unique terms in each paper and the system performance for extracting such terms.

**Table 6:** Unique term analysis for each paper.^a^

	synthesis papers								
	paper 1			paper 2			paper 3		
	uniq	extracted	coverage	uniq	extracted	coverage	uniq	extracted	coverage

SMaterial	15	8	0.53	6	5	0.83	16	10	0.63
MMethod	0	0	NA	0	0	NA	14	4	0.29
MChar	6	2	0.33	23	7	0.30	25	14	0.56
TArtifact	11	3	0.27	12	4	0.33	17	9	0.53
ExP	8	5	0.63	10	0	0.00	7	3	0.43
EvP	11	3	0.27	27	2	0.07	21	4	0.19
ExPVal	26	10	0.38	13	5	0.38	20	6	0.30
EvPVal	29	13	0.45	33	10	0.30	39	15	0.38
Total	106	44	0.42	124	33	0.27	159	65	0.41

	synthesis paper			characterization paper					
	paper 4			paper 5			corpus average coverage		
	uniq	extracted	coverage	uniq	extracted	coverage			
SMaterial	12	0	0.00	7	6	0.86	0.57		
MMethod	10	2	0.20	7	2	0.29	NA		
MChar	10	1	0.10	68	3	0.04	0.27		
TArtifact	13	2	0.15	46	4	0.09	0.28		
ExP	11	1	0.09	22	0	0.00	0.23		
EvP	52	11	0.21	49	17	0.35	0.22		
ExPVal	38	11	0.29	23	8	0.35	0.34		
EvPVal	44	10	0.23	52	9	0.17	0.31		
Total	190	38	0.20	274	49	0.18	0.29		

^a^uniq: number of unique terms in each paper; extracted: number of terms identified by NaDevEx; coverage: coverage percentage of unique terms identified.

For SMaterial, even though there are many terms that are unique to the test data, the system can identify such terms with a considerably higher coverage ratio than is obtained for other information categories. In most cases, those terms are identified as Chemical Named Entities and the system can generalize the training data by using the information that has been provided by the CNER tool, discussed earlier. For the parameters ExP and EvP, precision is good when the system can use parameter list to identify parameter-related terms. However, because of the insufficient coverage of parameter-related terms used in nanocrystal device development, recall of these parameters is worse than the results of human annotators.

**Figure 5:**
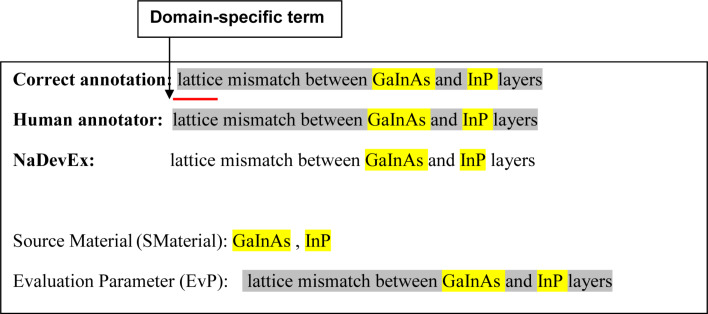
Domain-specific terms in NaDev corpus.

These results show that preprocessing annotation based on domain knowledge is generally promising, but coverage of the parameter information based on a list of physical quantities is not enough for nanocrystal device papers. As we have already discussed in the section “System performance analysis compared with human annotators”, there are many compound terms that contain particular domain-specific terms within their boundaries for characterizing categories. Figure 5 shows an example of such domain-specific terms.

Human annotators might be able to recognize such domain-specific terms with their domain knowledge. However, NaDevEx lacks such ability, specially with small training examples. It is necessary to evaluate the effectiveness of such a list by using a larger corpus.

### Discussion

The performance of NaDevEx is good for precision (95% for loose agreement overall), but is not good for recall (74% for loose agreement in total) at present. For the information category with rich domain-knowledge information (SMaterial), our system performance is almost comparable with that of human annotators. The precision of the system output is generally high: it is good (more than 95%) for MMethod, MChar, TArtifact and ExP but modest (more than 85%) for other categories (EvP, ExPVal, and EvPVal) with loose agreement. In contrast, the recall of the system is low (46–73%), even with loose agreement.

It is necessary to take into account the effect of the corpus size. As we discussed in Table 6, it is difficult to extract unique terms that do not exist in the training data (percentage of the unique terms among total terms is almost 30% (853/2870)). It is better to check the percentage of the unique terms among total terms when the size of the corpus increases. On the contrary, identification of non-unique terms is comparatively easier for such a small size corpus.

There are two possible research approaches to increase recall of the system output. One approach is to increase the corpus size. It is good to use one whole paper for clear understanding of the role of the terms in the paper, but the varieties of terms are not greatly increased because of the repetitive mention of terms. For the next step, it may be better to construct an abstract-based corpus to increase the variety of terms. It is also preferable to have a balanced mixture of synthesis and characterization papers. Another approach is to construct resources for representing domain knowledge. A list of terms that are frequently used in nanocrystal device papers is helpful to extract related terms that are in the list and variations of the terms based on the head terms in the list. There are physical parameters that cannot be extracted using the general physical quantities list (e.g., lattice, (111)B surface), so it is better to use vocabulary lists that include the parameters in this domain.

NaDevEx can be used as a preprocessor to find research papers that contain recent analysis results on nanocrystal devices to support the data collection process. Because NaDevEx is good at identifying source material, we can construct appropriate queries to restrict the output to papers that discuss a particular type of source material. Usage of other information categories may work well for finding related papers in a precision oriented manner, but it may miss papers because of the bad recall performance. A possible solution to this problem is implementing a framework that utilizes user-defined keyword lists as a knowledge resource for extracting such information. Another is using simple keyword search to find more papers that may contain such information.

## Conclusion

In this paper, we introduce NaDevEx, which automatically extracts useful information from nanocrystal device research papers based on the information categories defined in the NaDev corpus. This system has almost comparable performance with the human annotators for source material information, because of the good performance of the chemical named entity recognition system. For other categories, the precision is good (better than 85% in case of loose agreement), but there is a problem with recall because of the lack of examples, especially for characterization papers. To improve the performance, we discuss future research plans: increasing the corpus size by using abstract texts and constructing resources for representing domain knowledge (e.g., lists of parameters and manufacturing methods).
